# Auto-segmentation of cerebral cavernous malformations using a convolutional neural network

**DOI:** 10.1186/s12880-025-01738-6

**Published:** 2025-05-26

**Authors:** Chi-Jen Chou, Huai-Che Yang, Cheng-Chia Lee, Zhi-Huan Jiang, Ching-Jen Chen, Hsiu-Mei Wu, Chun-Fu Lin, I-Chun Lai, Syu-Jyun Peng

**Affiliations:** 1 Division of Neurosurgery, Department of Surgery, Kaohsiung Veterans General Hospital, Kaohsiung, Taiwan; 2 School of Medicine, College of Medicine, National Yang Ming Chiao Tung University, Taipei, Taiwan; 3 Department of Neurosurgery, Neurological Institute, Taipei Veterans General Hospital, Taipei, Taiwan; 4 Department of Electrical Engineering, National Central University, Taoyuan, Taiwan; 5 University of Texas Health Science Center at Houston, Houston, TX USA; 6 Department of Radiology, Taipei Veterans General Hospital, Taipei, Taiwan; 7 Department of Heavy Particles & Radiation Oncology, Taipei Veterans General Hospital, Taipei, Taiwan; 8 In-Service Master Program in Artificial Intelligence in Medicine, College of Medicine, Taipei Medical University, No.250, Wuxing St., Xinyi Dist, Taipei, 110 Taiwan; 9 Clinical Big Data Research Center, Taipei Medical University Hospital, Taipei Medical University, Taipei, Taiwan

**Keywords:** Gamma knife (GK) treatment planning, Cerebral cavernous malformations, Deep learning, Mask region-based convolutional neural network, DeepMedic

## Abstract

**Background:**

This paper presents a deep learning model for the automated segmentation of cerebral cavernous malformations (CCMs).

**Methods:**

The model was trained using treatment planning data from 199 Gamma Knife (GK) exams, comprising 171 cases with a single CCM and 28 cases with multiple CCMs. The training data included initial MRI images with target CCM regions manually annotated by neurosurgeons. For the extraction of data related to the brain parenchyma, we employed a mask region-based convolutional neural network (Mask R-CNN). Subsequently, this data was processed using a 3D convolutional neural network known as DeepMedic.

**Results:**

The efficacy of the brain parenchyma extraction model was demonstrated via five-fold cross-validation, resulting in an average Dice similarity coefficient of 0.956 ± 0.002. The segmentation models used for CCMs achieved average Dice similarity coefficients of 0.741 ± 0.028 based solely on T2W images. The Dice similarity coefficients for the segmentation of CCMs types were as follows: Zabramski Classification type I (0.743), type II (0.742), and type III (0.740). We also developed a user-friendly graphical user interface to facilitate the use of these models in clinical analysis.

**Conclusions:**

This paper presents a deep learning model for the automated segmentation of CCMs, demonstrating sufficient performance across various Zabramski classifications.

**Trial registration:**

not applicable.

**Supplementary Information:**

The online version contains supplementary material available at 10.1186/s12880-025-01738-6.

## Introduction

Cerebral cavernous malformations (CCMs), one of the four types of cerebral vascular malformations—along with arteriovenous malformations, developmental venous anomalies, and capillary telangiectasia—account for approximately 10–15% of all such vascular anomalies [1, 2]. Due to their low internal blood pressure, CCMs are not visible in angiographic images [3]; however, they exhibit characteristic patterns on MRI, due largely to surrounding hemosiderin deposits resulting from repeated microhemorrhages [4]. Zabramski et al. proposed a classification system for CCMs based on MRI features, focusing on patterns of hemorrhagic and hemosiderin deposition [5].

Many CCM patients experience seizures [6], with a mean annual incidence of 1.5–2.4% [7]. Cortical involvement and lesion location have been identified as risk factors for seizure occurrence [7, 8]. In a previous study, we detected a significant correlation between seizure prevalence and the CCM-induced displacement of gray matter [9]. However, manual delineation of lesion regions during treatment planning is labor-intensive and highly dependent on operator experience.

Recent advances in deep learning, particularly convolutional neural networks (CNNs), have enabled automated segmentation of brain lesions [10–13]. However, applying these techniques to CCMs remains challenging due to their diverse imaging presentations.

In the current study, we developed a deep learning model for the automated segmentation of CCMs using unprocessed MRI data. Our objective was to facilitate lesion delineation in Gamma Knife (GK) radiosurgery and related clinical research, while improving the consistency of segmentation results.

## Materials and methods

### Subjects

This study was based on GK data from 197 patients with single or multiple CCMs collected from Taipei Veterans General Hospital between February 2004 and August 2022. Five of the patients were excluded, due to the presence of additional meningioma *(n* = 3) or peritumoral edema (*n* = 2), which could have affected the CCM segmentation model. Six of the patients underwent repeated GK treatment due to symptom progression. This left a total of 192 patients and 199 exams, including 171 exams with a single CCM (167 patients) and 28 exams with multiple CCMs (25 patients). The ROIs were delineated by neurosurgeons during GK planning. Lesions that were not outlined in GK planning were manually delineated and added. The overall average CCM volume was 2.805 ± 3.455 ml. In cases with a single CCM, the average volume was 2.805 ± 3.455 ml (ranging = 0.046 to 19.421 ml). In cases with multiple CCMs, the average volume was 4.137 ± 3.782 ml (ranging = 0.478 to 16.344 ml). The study was approved by the Institutional Review Board (2024-06-025CC).

### MRI protocol

MRI images were acquired using a Signa HDxt™ (GE Medical Systems). T2W and T1-weighted images with contrast enhancement (T1WIC) were used for model training. The protocol used in the capture of T2W sequences was as follows: repetition time (TR) = 3233.34 ms, echo time (TE) = 99.52-109.472 ms, magnetic field strength = 1.5 T, slice thickness = 3 mm, spacing between slices = 3 mm, flip angle = 90°, and voxel size = 0.5078 × 0.5078 × 3 mm³. The protocol used in the capture of T1WIC sequences was as follows: TR = 433.3–500 ms, TE = 8–9 ms, magnetic field strength = 1.5 T, slice thickness = 3 mm, spacing between slices = 3 mm, flip angle = 90°, and voxel size = 0.5078 × 0.5078 × 3 mm³.

### Deep learning model

**Fig. 1:**
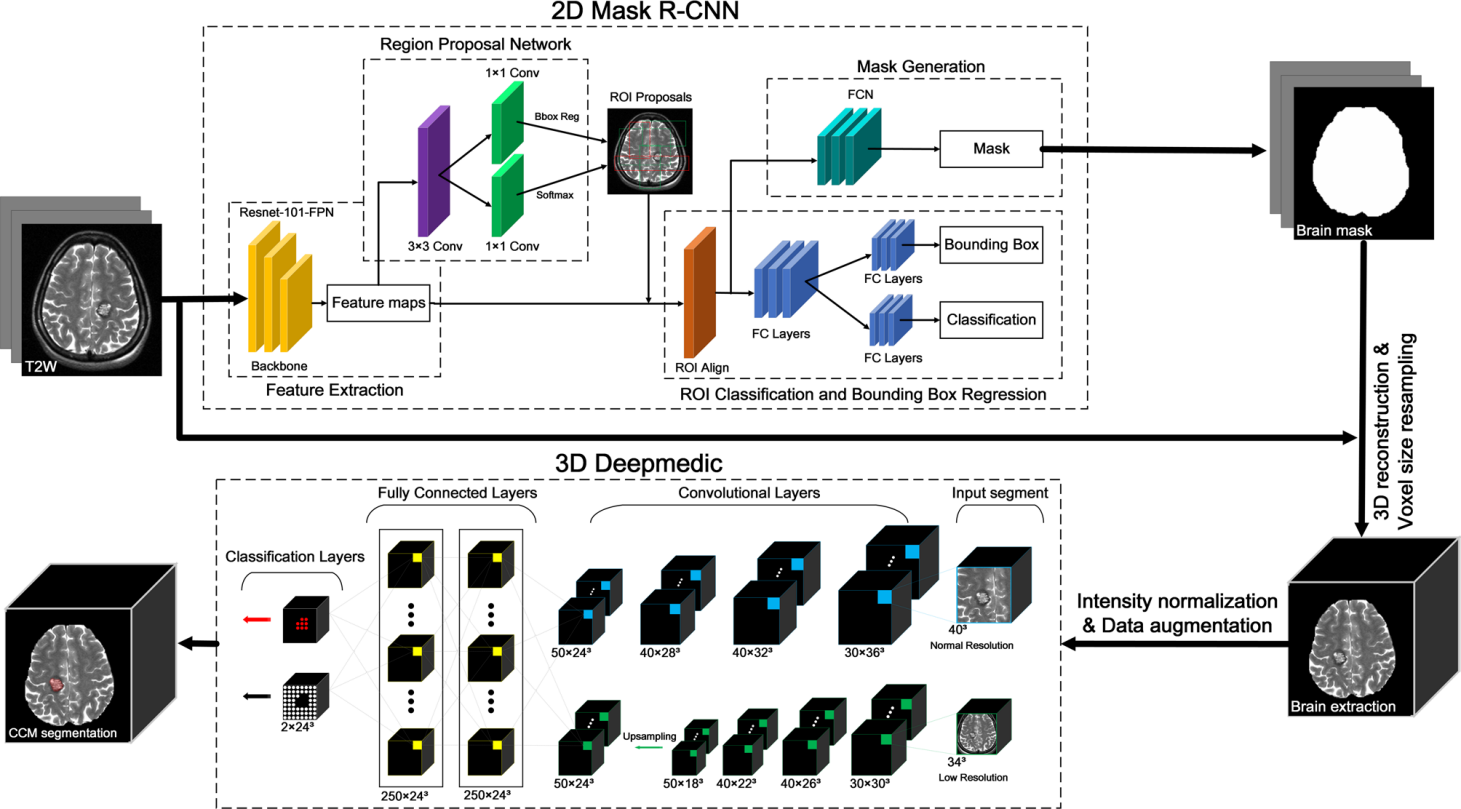
Flowchart of proposed CCM auto-segmentation process

As shown in Fig. 1, the preprocessing of T2W images involved brain extraction, voxel resampling, and intensity normalization. The process of brain extraction to define the ROIs was performed using the Mask RCNN model in 2D, whereas CCM segmentation and quantification were performed using the DeepMedic CNN in 3D. This process was implemented on a computer with an AMD Ryzen 7 5800 H processor (3.20 GHz), 16 GB of RAM, and a Radeon Graphics card as well as a dedicated Nvidia GeForce GPU RTX 3060 with 6 GB of VRAM. Computation was performed in an Anaconda virtual environment (set up using Python 3.7) in conjunction with imported deep learning libraries (TensorFlow-gpu 2.5 and Keras 2.5).

#### Step 1 Extraction of brain parenchyma

Extraction of the brain parenchyma involved adjusting the orientation of the original T2W images to align it with the anterior commissure (AC) of the individual brain space. Brain extraction was then performed to remove signals associated with the skull and scalp from the original images. The Statistical Parametric Mapping 12 (SPM12) (https://www.fil.ion.ucl.ac.uk/spm/software/spm12/) tool was used to derive the brain parenchyma, the results of which were verified or adjusted by physicians to establish a gold standard.

A 2D neural network (Mask R-CNN) was used to train a model for the automated extraction of parenchymal brain from each scan (Supplementary Fig. 1) [14]. Note that a total of 108 scans were used for brain extraction, with 90 scans subjected to 5-fold cross-validation (72 for training and 18 for validation) and 18 scans used as a test set to evaluate the performance of the trained segmentation model. We also augmented the training dataset by dividing the 108 scans into six equal sections. Four sections were used as the training set, one was used as the validation set, and one was used as the testing set.

#### Step 2 CCM segmentation

Data preprocessing is required to deal with variations in voxel size and grayscale intensity due to differences among MRI instruments and scanning times. Prior to inputting into the 3D CNN, the MRI images were resampled by scaling all voxels in each image from an initial size of 0.508 × 0.508 × 3.01 mm³ to 1 × 1 × 1 mm³. Z-score intensity normalization was then applied to standardize the grayscale intensity of each data point, resulting in a mean of 0 and a standard deviation of 1.

Data augmentation was used to enhance the CCM segmentation ability of the neural network and prevent overfitting. This involved generating additional images with random rotations along the X, Y, and Z axes (-45 to 45 degrees) and random scaling (0.9 to 1.1 times).

CCM segmentation performance was evaluated using the aforementioned dataset as well as a second dataset comprising 84 cases, for a total of 192 cases. The above-mentioned data were split into five equal subsets, with three subsets assigned to the training set, one assigned to the validation set, and one assigned to the testing set.

After extracting a brain mask from T2W images, we conducted a series of preprocessing steps aimed at improving CCM segmentation performance. The neural network employed for this task was DeepMedic [15], which is a multi-scale 3D CNN consisting of convolutional layers, fully connected layers, and classification layers (see Supplementary Fig. 2).

A key feature of this neural network is its dual-channel design, in which the first channel extracts normal-scale image patches (to capture features in detail) and the second channel extracts large-scale image patches (to capture spatial positional information). Utilizing separate channels for the extraction of image feature patches at multiple resolutions enhances the ability of DeepMedic to learn to recognize CCMs of various sizes in various brain locations. Note that after both channels complete convolutional processing in the fully convolutional layers, the second channel undergoes up-sampling to facilitate processing in fully connected layers for information matching and classification. The final classification layer outputs the CCM segmentation results.

The performance of the model in brain extraction and CCM segmentation was evaluated using several metrics. The binary mask predicted by the model was compared with gold standard values, yielding true positive (TP), false positive (FP), true negative (TN), and false negative (FN) values, based on a confusion matrix. Performance was also evaluated in terms of Dice coefficient, Precision, and Recall.

## Results

### Demography

The average age of the 192 patients at the time of their examination was 41.8 ± 14.0 years. Seizures were reported in 28 patients (29 exams), and episodes of hemorrhage were reported in 174 patients (181 exams). This study addressed a total of 199 exams, including cases with a single CCM (*n* = 171) and those with multiple CCMs (*n* = 28). The average CCM volume was 2.02 ± 2.60 ml. Table 1 lists the locations of the CCMs and additional information.

**Table 1:** Demographic data

**Patient demographic data**	
Number of cases	192 patients (199 exams)
Age at time of exam (y)	41.8 ± 14.0 (9.8–79.7)
Gender (M: F)	91:108
Cases involving seizure	28 patients (29 exams)
Cases involving hemorrhage	174 patients (181 exams)
**Image demographics**	
Total number of CCM lesions Single-lesion cases Multi-lesion cases	199 exams (247 lesions) 171 28 exams (76 lesions)
Region Frontal Parietal Temporal Occipital Brainstem Cerebellum Other	Number of lesions (M*) 45 (M19) 14 (M6) 24 (M9) 5 (M4) 94 (M17) 27 (M12) 38 (M9)
Mean CCM volume	2.02 ± 2.60 ml (0.03-14.4 ml)

CCM: cerebral cavernous malformation

*M: number of lesions from multiple-lesion cases

### Brain extraction

**Fig. 2:**
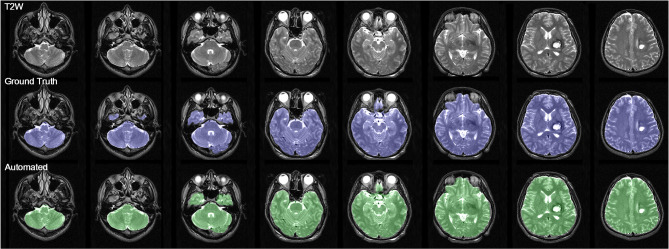
Brain extraction results with a dice coefficient of 0.968, precision of 0.993, and recall of 0.944

During the brain extraction stage, 5-fold cross-validation was conducted using 108 scans. The evaluation data were divided into six equal parts, with four parts used for training, one part used for validation, and one part for testing. The 5-fold cross-validation results in Supplementary Table 1 show that the Dice coefficient consistently exceeded 0.95 with average precision of 0.987 and average recall values of 0.924. Among the folds, the model trained in the second fold consistently outperformed those trained in the other folds. We therefore adopted the model from the second fold for brain extraction across all scans. In terms of brain extraction performance, this model achieved a Dice coefficient of 0.968 with precision of 0.993 and recall of 0.944 (see Fig. 2).

### CCM segmentation

**Fig. 3:**
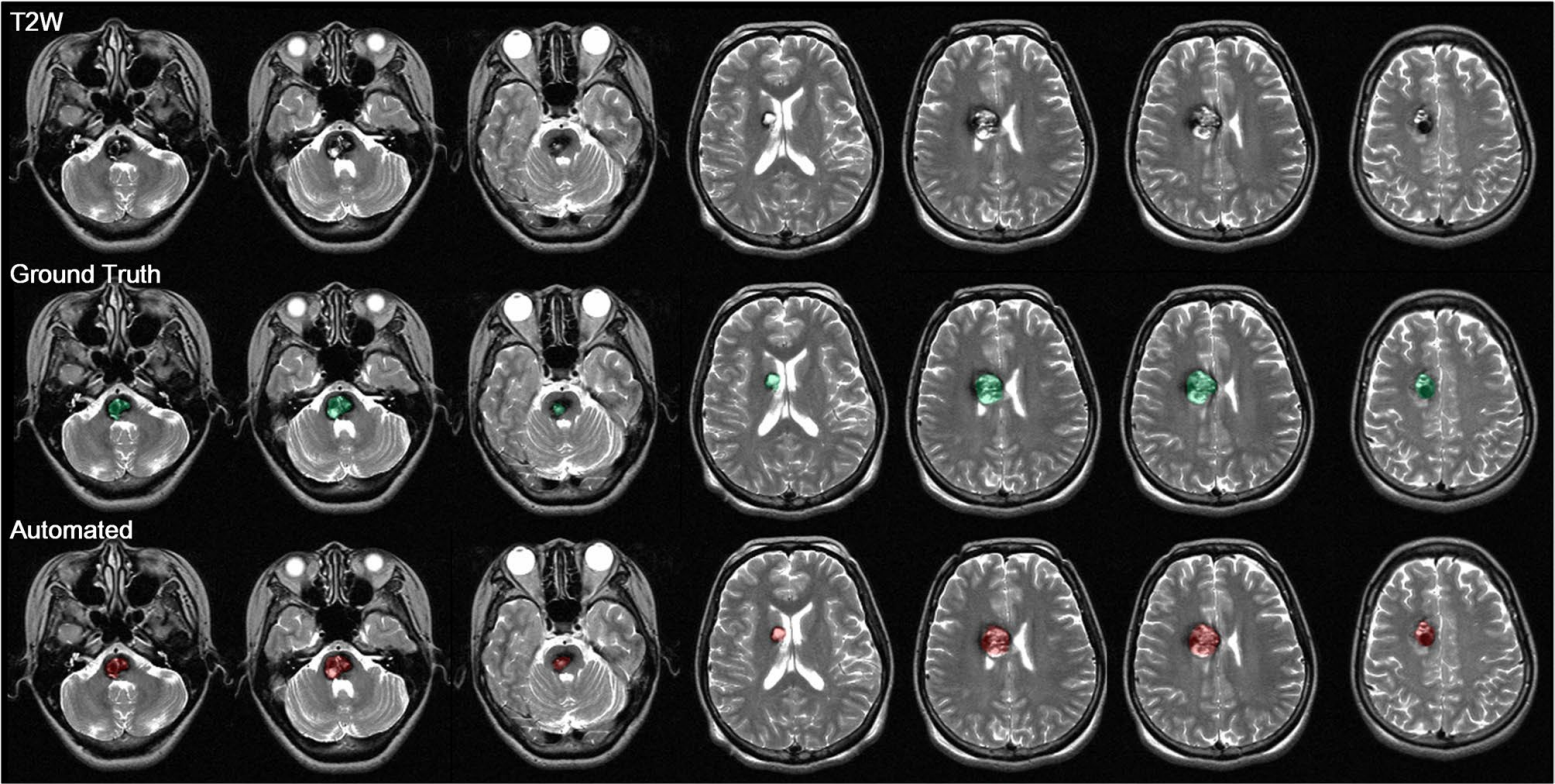
Illustrative case of deep learning-based auto-segmentation: (first row) T2W training input; (second row) ground truth delineated manually; (third row) automated CCM segmentation results. Performance metrics: Precision (0.892), recall (0.866), and Dice similarity index (0.879)

In the brain extraction stage, 5-fold cross-validation was conducted using a dataset comprising 192 scans. The scans were evenly distributed across folds based on CCM volume. Note that the 192 scans included 25 scans containing multiple CCMs, which were also evenly distributed among the five cross-validation groups. Paired t-tests were conducted to assess differences in segmentation performance between the two models (T2W alone vs. T2W plus T1WIC). Our analysis revealed no statistically significant differences between the two models in terms of Dice coefficient (0.741 ± 0.028 vs. 0.754 ± 0.03, *p* = 0.084), precision (0.806 ± 0.013 vs. 0.797 ± 0.024, *p* = 0.127), or recall (0.735 ± 0.032 vs. 0.762 ± 0.058, *p* = 0.063) (Supplementary Table 2). Thus, it appears that the inclusion of T1WIC images did not substantially improve segmentation performance. Figure 3, Supplementary Fig. 3, and Supplementary Fig. 4 present illustrative examples of deep learning-based auto-segmentation.

### CCM subgroup analysis in different Zabramski types

The characteristics of hemorrhage and hemosiderin deposition vary among the various types of CCM. In accordance with Zabramski classification, the CCMs in this study were categorized manually into four subgroups: type 1 (39 lesions), type II (73 lesions), type III (63 lesions), and type IV (2 lesions) [5]. Table 2 lists the performance parameters of the model trained using T2W images alone. Using this model, the Dice coefficients for each type were as follows: type I (0.743 ± 0.178; 95% confidence interval (CI): 0.685–0.801), type II (0.742 ± 0.147; 95% CI: 0.707–0.776), type III (0.740 ± 0.165; 95% CI: 0.698–0.781), and type IV (0.361 ± 0.315). Using the same model, the precision was as follows: type I (0.771 ± 0.200; 95% CI: 0.706–0.836), type II (0.827 ± 0.319; 95% CI: 0.794–0.859), type III (0.813 ± 0.190; 95% CI: 0.765–0.861), and type IV (0.617 ± 0.542). Using the same model, the recall was as follows: type I (0.787 ± 0.202; 95% CI: 0.721–0.852), type II (0.713 ± 0.192; 95% CI: 0.668–0.758), type III (0.737 ± 0.185; 95% CI: 0.690–0.783), and type IV (0.255 ± 0.221).

## Discussion

This study developed a deep learning model for the automated segmentation of CCMs using T2W MRI images. Our results demonstrated the feasibility of this approach, achieving satisfactory segmentation performance across various Zabramski types. The inclusion of T1WIC with T2W images during training led to modest improvements in some metrics; however, overall performance was hindered by a notable drop in precision. Thus, the final model was trained using only T2W images.

Subgroup analysis revealed similar Dice coefficients for Zabramski types I, II, and III, indicating consistent segmentation performance. However, the model performed poorly when applied to type IV lesions, likely due to subtle imaging features and low visibility on conventional MRI scans. This finding aligns with the clinical characteristics of type IV CCMs, which are often occult and asymptomatic, leading to their underrepresentation in the dataset.

Further analysis of missegmentation cases revealed that model performance was influenced by lesion characteristics, such as size, occurrence depth, and lesion count. Small lesions were frequently missed (false negatives), and those in complex anatomical regions (e.g., the brainstem or basal ganglia) were difficult to detect due to low image contrast and/or spatial distortion. In some cases, multiple adjacent CCMs led to over-segmentation. Although developmental venous anomalies (DVAs) were explicitly excluded from the ROIs during manual delineation, adjacent vascular structures may have introduced ambiguity during manual delineation.

This work represents one of the first attempts to apply deep learning for CCM auto-segmentation. Wang et al. [16] previously applied machine learning for lesion detection; however, their approach lacked full auto-segmentation capability and relied heavily on manual input. In contrast, out approach leverages convolutional neural networks to learn imaging features directly from raw MRI data, providing a more robust and scalable solution. Nevertheless, accurate segmentation of CCM in subgroups remains challenging, particularly in subgroups with atypical imaging presentations.

This study also has several limitations. The derivation of our dataset from GK treatment planning data may have introduced selection bias toward lesions that were small, deep-seated, or located in eloquent brain regions. As a result, large or hemorrhagic lesions were underrepresented. Moreover, the limited number of cases involving multiple-lesion CCMs and Zabramski type IV lesions prevented the training of separate models or detailed performance evaluations for these subgroups. Future studies with larger, more balanced datasets would enable subgroup-specific analysis and improve model generalizability. Such efforts could facilitate clinical workflows in radiosurgery planning and related research; however, further efforts are needed to enhance model performance, particularly for complex lesions.

## Conclusions

This paper presents a deep learning model for the automated segmentation of CCMs using T2W images, demonstrating reliable performance across various multiple Zabramski classifications. The proposed scheme represents an important step in reducing manual workload and increasing consistency in lesion delineation, with potential applications in clinical and research settings.

**Table 2:** Segmentation performance of model trained using T2W images when applied to CCM types based on Zabramski classification

Zabramski classification	I (*n* = 39)	II (*n* = 73)	III (*n* = 63)	IV (*n* = 2)
DICE	0.743 ± 0.178 (0.685–0.801)	0.742$\:\:\pm\:\:0.147\:$ (0.707–0.776)	0.740 ± 0.165 (0.698–0.781)	0.361 ± 0.315
Precision	0.771 ± 0.200 (0.706–0.836)	0.827$\:\:\pm\:\:0.319$ (0.794–0.859)	0.813 ± 0.190 (0.765–0.861)	0.617 ± 0.542
Recall	0.787 ± 0.202 (0.721–0.852)	0.713 ± 0.192 (0.668–0.758)	0.737 ± 0.185 (0.690–0.783)	0.255 ± 0.221

Mean ± standard deviation (95% confidence interval)

## Electronic supplementary material

Below is the link to the electronic supplementary material.

Supplementary Material 1

Supplementary Material 2

Supplementary Material 3

Supplementary Material 4

Supplementary Material 5

Supplementary Material 6

Supplementary Material 7

Supplementary Material 8

Supplementary Material 9

## Data Availability

The datasets used and/or analyzed during the current study are available from the corresponding author on reasonable request.

## Abbreviations

CCMs: Cerebral cavernous malformations
CNNs: Convolutional neural networks
GK: Gamma Knife
Mask R-CNN: Mask region-based convolutional neural network
ROIs: Regions of interest
SPM12: Statistical Parametric Mapping 12

## References

[1] McCormick WF. The pathology of vascular (Arteriovenous) malformations. J Neurosurg. 1966;24(4):807–16. 5934138 10.3171/jns.1966.24.4.0807

[2] Batra S, Lin D, Recinos PF, Zhang J, Rigamonti D. Cavernous malformations: natural history, diagnosis and treatment. Nat Reviews Neurol. 2009;5(12):659–70. 10.1038/nrneurol.2009.177 19953116

[3] Dalyai RT, Ghobrial G, Awad I, Tjoumakaris S, Gonzalez LF, Dumont AS, et al. Management of incidental cavernous malformations: a review. Neurosurgical Focus FOC. 2011;31(6):E5. 10.3171/2011.9.FOCUS11211 22133177

[4] Raychaudhuri R, Batjer HH, Awad IA. Intracranial cavernous Angioma: a practical review of clinical and biological aspects. Surg Neurol. 2005;63(4):319–28. 15808709 10.1016/j.surneu.2004.05.032

[5] Zabramski JM, Wascher TM, Spetzler RF, Johnson B, Golfinos J, Drayer BP, et al. The natural history of Familial cavernous malformations: results of an ongoing study. J Neurosurg. 1994;80(3):422–32. 8113854 10.3171/jns.1994.80.3.0422

[6] Rosenow F, Alonso-Vanegas MA, Baumgartner C, Blümcke I, Carreño M, Gizewski ER, et al. Cavernoma‐related epilepsy: review and recommendations for management—Report of the surgical task force of the ILAE commission on therapeutic strategies. Epilepsia. 2013;54(12):2025–35. 24134485 10.1111/epi.12402

[7] Menzler K, Chen X, Thiel P, Iwinska-Zelder J, Miller D, Reuss A, et al. Epileptogenicity of Cavernomas depends on (Archi-) cortical localization. Neurosurgery. 2010;67(4):918–24. 20881556 10.1227/NEU.0b013e3181eb5032

[8] Casazza M, Broggi G, Franzini A, Avanzini G, Spreafico R, Bracchi M, et al. Supratentorial cavernous Angiomas and epileptic seizures: preoperative course and postoperative outcome. Neurosurgery. 1996;39(1):26–34. 8805137 10.1097/00006123-199607000-00007

[9] Chou C-J, Lee C-C, Chen C-J, Yang H-C, Peng S-J. Displacement of Gray matter and incidence of seizures in patients with cerebral cavernous malformations. Biomedicines. 2021;9(12):1872. 34944688 10.3390/biomedicines9121872 PMC8698264

[10] Işın A, Direkoğlu C, Şah M. Review of MRI-based brain tumor image segmentation using deep learning methods. Procedia Comput Sci. 2016;102:317–24.

[11] Ghaffari M, Sowmya A, Oliver R. Automated brain tumor segmentation using multimodal brain scans: a survey based on models submitted to the brats 2012–2018 challenges. IEEE Rev Biomed Eng. 2019;13:156–68. 31613783 10.1109/RBME.2019.2946868

[12] Urban G, Bendszus M, Hamprecht F, Kleesiek J. Multi-modal brain tumor segmentation using deep convolutional neural networks. MICCAI BraTS (brain tumor segmentation) challenge Proceedings, winning contribution. 2014:31– 5.

[13] Ranjbarzadeh R, Bagherian Kasgari A, Jafarzadeh Ghoushchi S, Anari S, Naseri M, Bendechache M. Brain tumor segmentation based on deep learning and an attention mechanism using MRI multi-modalities brain images. Sci Rep. 2021;11(1):10930. 34035406 10.1038/s41598-021-90428-8 PMC8149837

[14] He K, Gkioxari G, Dollár P, Girshick R, Mask R-CNN. IEEE Trans Pattern Anal Mach Intell. 2020;42(2):386–97. 29994331 10.1109/TPAMI.2018.2844175

[15] Kamnitsas K, Ledig C, Newcombe VFJ, Simpson JP, Kane AD, Menon DK, et al. Efficient multi-scale 3D CNN with fully connected CRF for accurate brain lesion segmentation. Med Image Anal. 2017;36:61–78. 27865153 10.1016/j.media.2016.10.004

[16] Wang H, Ahmed SN, Mandal M. Computer-aided diagnosis of cavernous malformations in brain MR images. Comput Med Imaging Graph. 2018;66:115–23. 29609039 10.1016/j.compmedimag.2018.03.004

