# Supplementary material for: Auto-segmentation of cerebral cavernous malformations using a convolutional neural network
Source: BMC Med Imaging. 2025 May 26;25:190. doi: 10.1186/s12880-025-01738-6 (PMC12107882; doi:10.1186/s12880-025-01738-6)
Supplement: Supplementary file 1 — Supplementary Material 1 [file 12880_2025_1738_MOESM1_ESM.docx]

**Supplemental information**

**Supplementary Figure 1** Structure of Mask R-CNN for brain parenchyma extraction.

**Supplementary Figure 2** Structure of DeepMedic model for CCM segmentation.

**Supplementary Figure 3** Illustrative case of deep learning-based auto-segmentation: (first row) T2W training input; (second row) ground truth delineated manually; (third row) automated CCM segmentation results. Performance metrics: Precision (0.911), recall (0.911), and Dice similarity index (0.911).

**Supplementary Figure 4** Illustrative case of deep learning-based auto-segmentation: (first row) T2W training input; (second row) ground truth delineated manually; (third row) automated CCM segmentation results. Performance metrics: Precision (0.891), recall (0.889), and Dice similarity index (0.891).

**Supplementary Figure 5** A user-friendly graphical user interface for CCMs that automates the calculation of lesion volume and number.

**Supplementary Table 1** Results of 5-fold cross-validation for brain extraction.

**Supplementary Table 2** Cross-validation results for CCM segmentation: T2W alone vs. T2W plus T1WIC.

**Supplementary Video 1** Steps for using the graphical user interface.
