# Supplementary figures and images for: Auto-segmentation of cerebral cavernous malformations using a convolutional neural network
Source: BMC Med Imaging. 2025 May 26;25:190. doi: 10.1186/s12880-025-01738-6 (PMC12107882; doi:10.1186/s12880-025-01738-6)

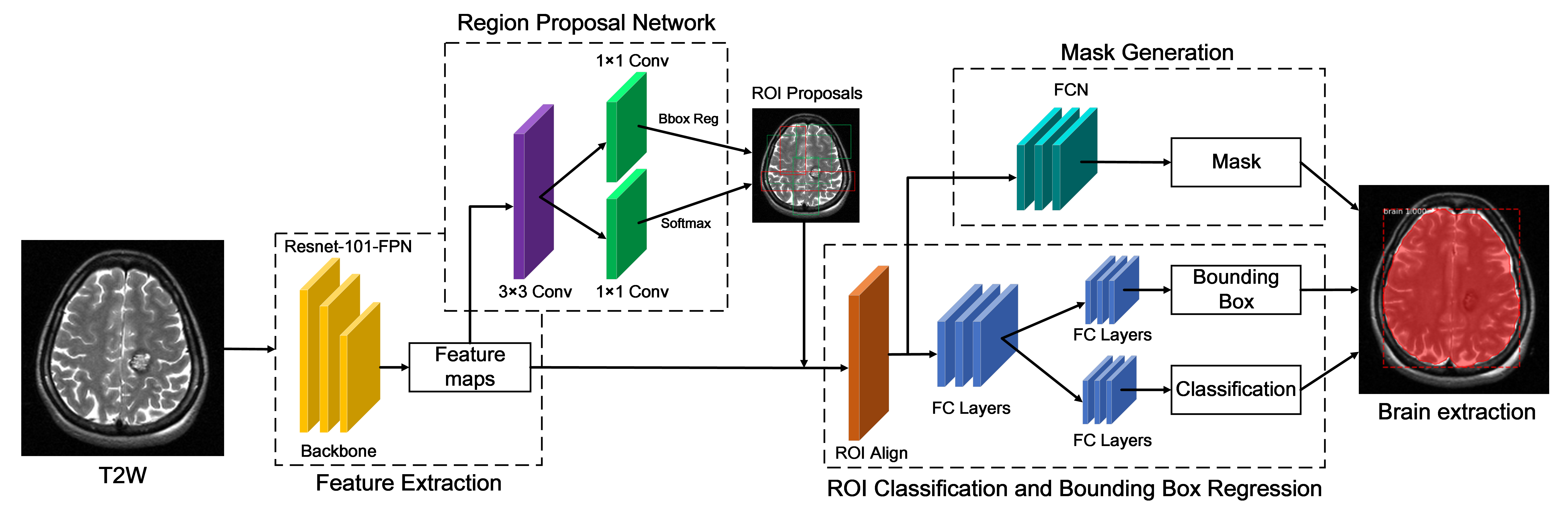

Supplement: Supplementary file 2 — Supplementary Material 2 [file 12880_2025_1738_MOESM2_ESM.tif]

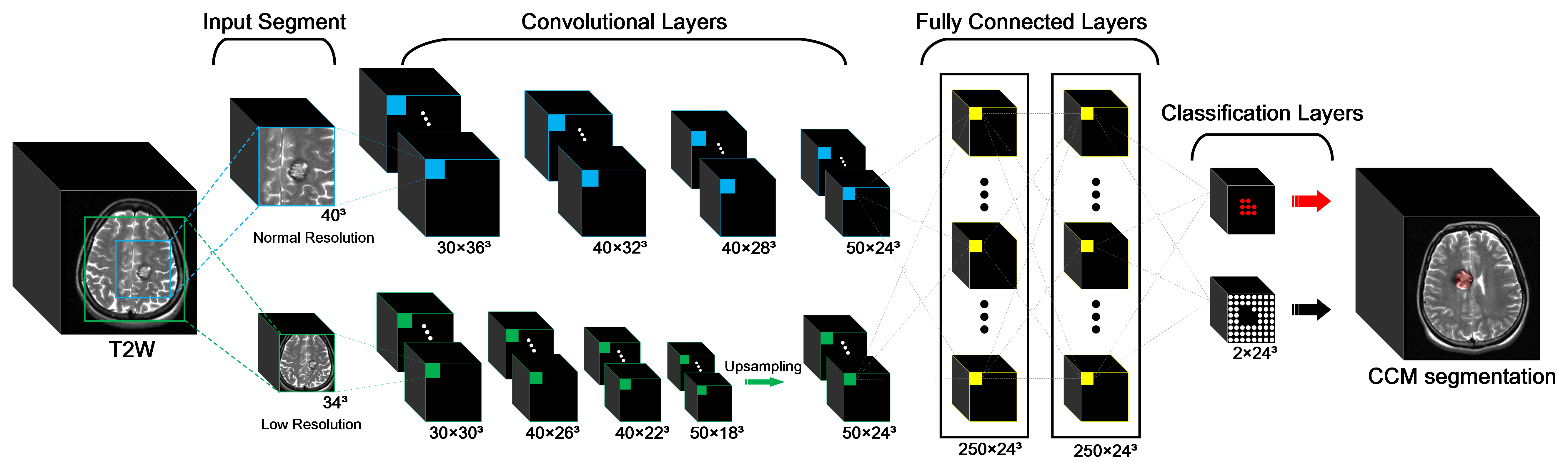

Supplement: Supplementary file 3 — Supplementary Material 3 [file 12880_2025_1738_MOESM3_ESM.tif]

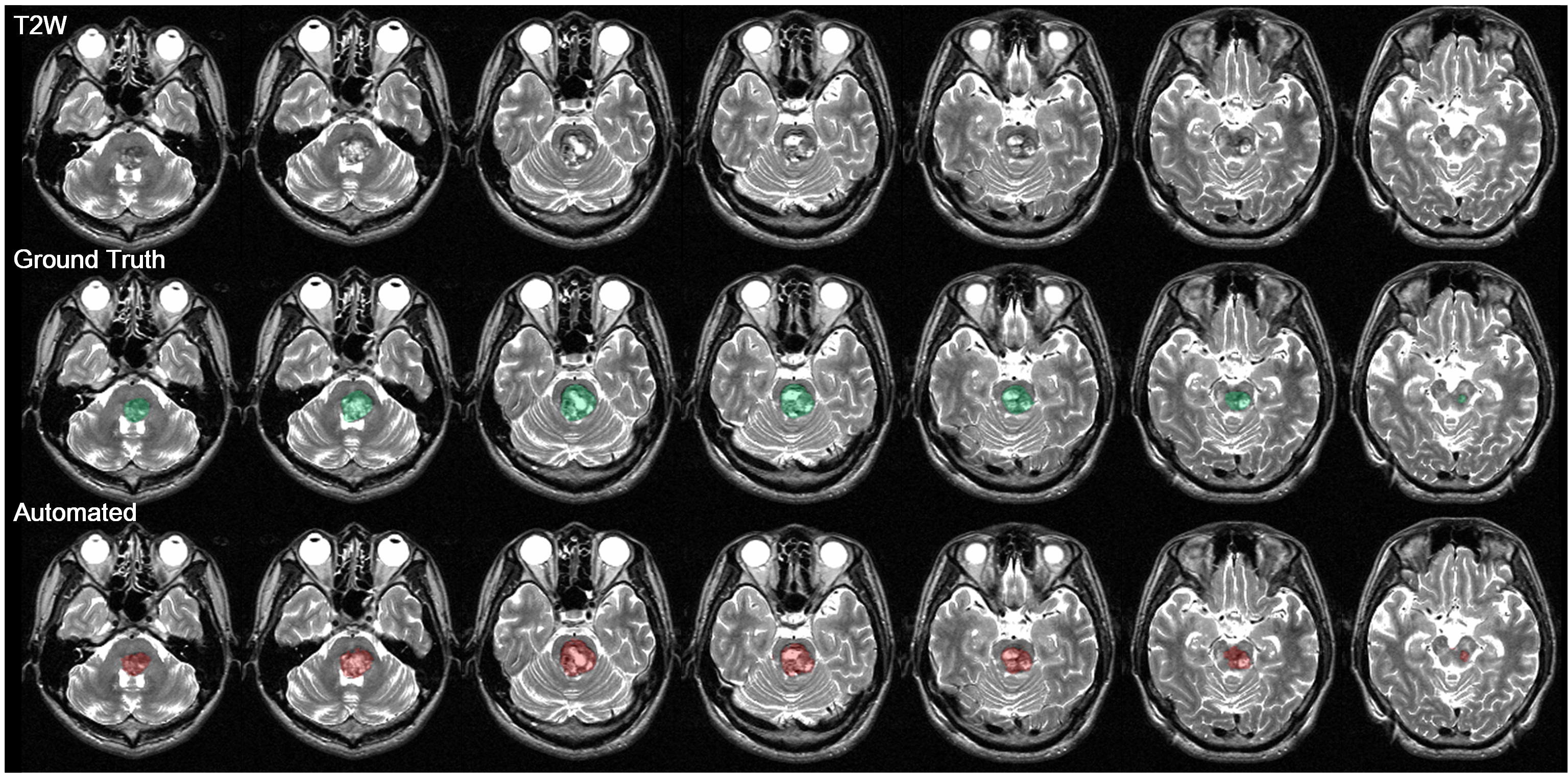

Supplement: Supplementary file 4 — Supplementary Material 4 [file 12880_2025_1738_MOESM4_ESM.tif]

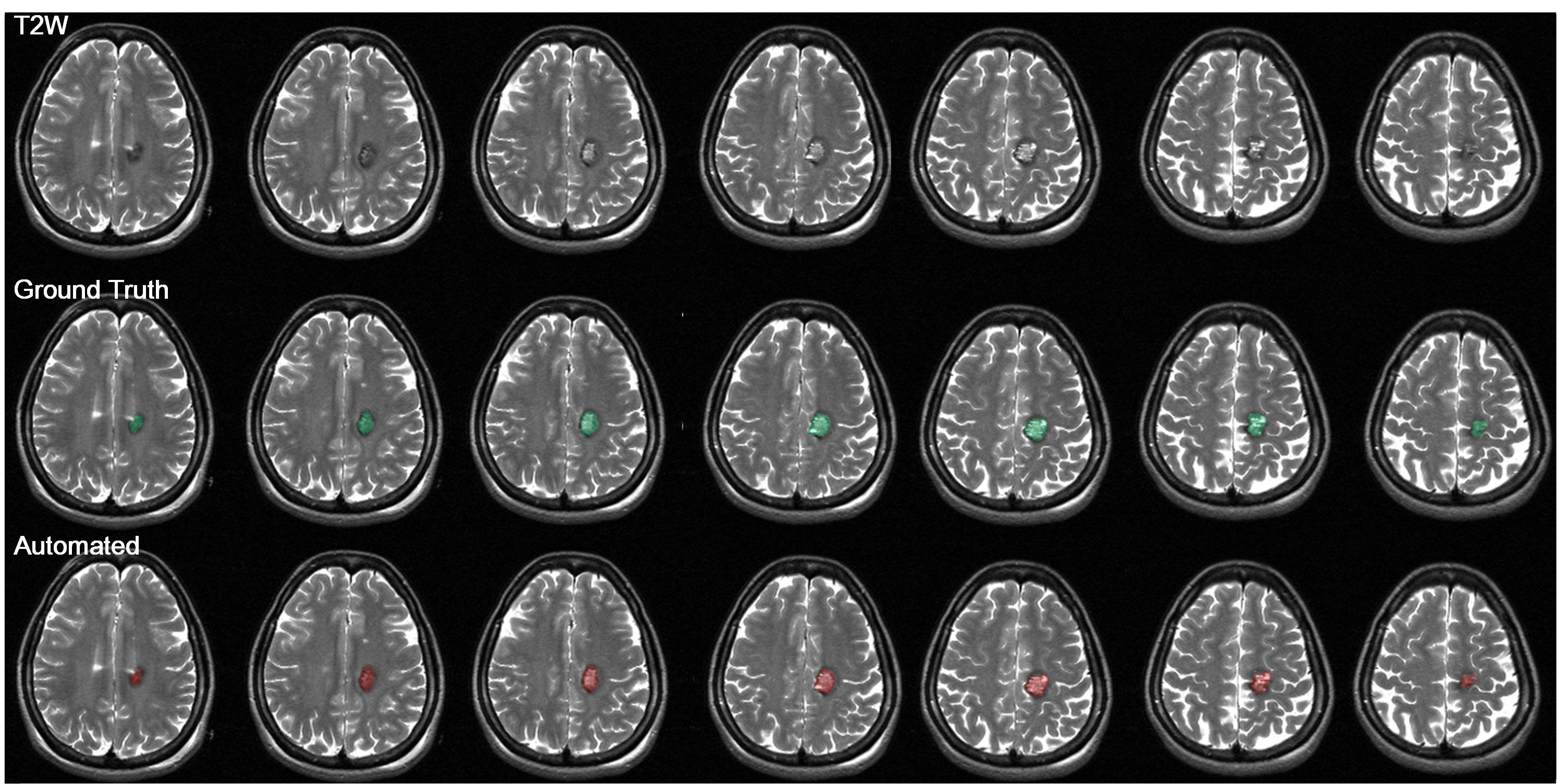

Supplement: Supplementary file 5 — Supplementary Material 5 [file 12880_2025_1738_MOESM5_ESM.tif]

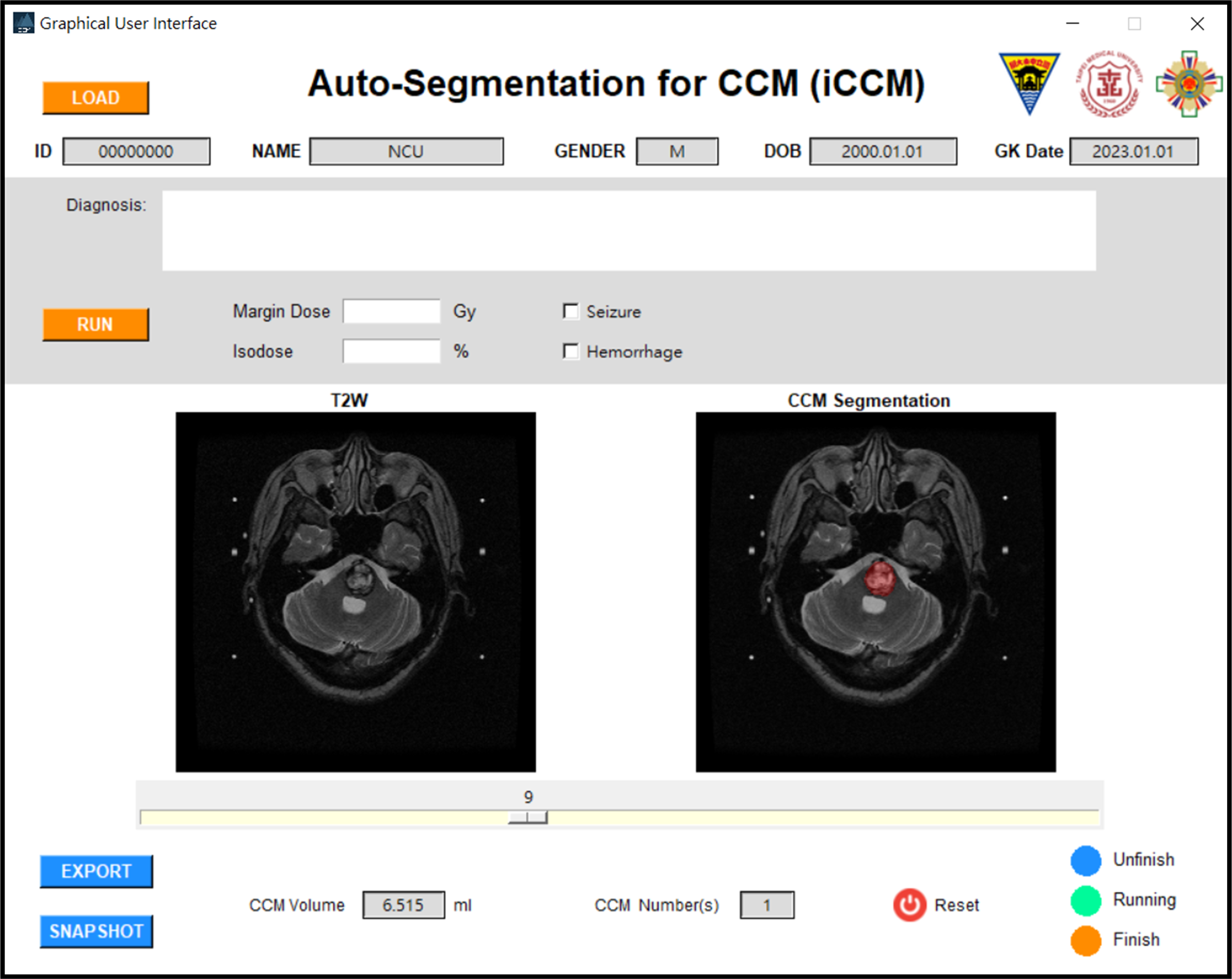

Supplement: Supplementary file 6 — Supplementary Material 6 [file 12880_2025_1738_MOESM6_ESM.tif]
