# Supplementary material for: Auto-segmentation of cerebral cavernous malformations using a convolutional neural network
Source: BMC Med Imaging. 2025 May 26;25:190. doi: 10.1186/s12880-025-01738-6 (PMC12107882; doi:10.1186/s12880-025-01738-6)
Supplement: Supplementary file 7 — Supplementary Material 7 [file 12880_2025_1738_MOESM7_ESM.docx]

Supplementary Table 1 Results of 5-fold cross-validation for brain extraction.

| Fold | Dice | Precision | Recall |
| --- | --- | --- | --- |
| 1 | 0.956 | 0.987 | 0.911 |
| 2 | 0.959 | 0.988 | 0.931 |
| 3 | 0.957 | 0.988 | 0.929 |
| 4 | 0.953 | 0.986 | 0.922 |
| 5 | 0.956 | 0.987 | 0.928 |
| Mean ± STD | 0.956 ± 0.002 | 0.987 ± 0.001 | 0.924 ± 0.008 |

STD: standard deviation.
