# Supplementary material for: Auto-segmentation of cerebral cavernous malformations using a convolutional neural network
Source: BMC Med Imaging. 2025 May 26;25:190. doi: 10.1186/s12880-025-01738-6 (PMC12107882; doi:10.1186/s12880-025-01738-6)
Supplement: Supplementary file 8 — Supplementary Material 8 [file 12880_2025_1738_MOESM8_ESM.docx]

Supplementary Table 2 Cross-validation results for CCM segmentation: T2W alone vs. T2W plus T1WIC.

| Fold | Dice | | Precision | | Recall | |
| --- | --- | --- | --- | --- | --- | --- |
|  | T2W | T2W + T1WIC | T2W | T2W + T1WIC | T2W | T2W + T1WIC |
| 1 | 0.732 | 0.752 | 0.799 | 0.762 | 0.738 | 0.788 |
| 2 | 0.769 | 0.796 | 0.819 | 0.802 | 0.758 | 0.825 |
| 3 | 0.742 | 0.748 | 0.822 | 0.787 | 0.721 | 0.769 |
| 4 | 0.696 | 0.712 | 0.791 | 0.827 | 0.689 | 0.668 |
| 5 | 0.759 | 0.763 | 0.801 | 0.805 | 0.771 | 0.761 |
| Mean± STD | 0.741 ± 0.028 | 0.754 ± 0.03 | 0.806 ± 0.013 | 0.797 ± 0.024 | 0.735 ± 0.032 | 0.762 ± 0.058 |
| *p* | 0.084 | | 0.127 | | 0.063 | |

T2W : T2-weighted images, T1WIC: T1-weighted images with contrast enhancement, STD: standard deviation.
